# Statistical analysis for explosives detection system test and evaluation

**DOI:** 10.1038/s41598-021-03755-1

**Published:** 2022-01-07

**Authors:** Stefan Lukow, James C. Weatherall

**Affiliations:** Present Address: U. S. Department of Homeland Security, Science and Technology Directorate, Transportation Security Laboratory, W. J. Hughes Technical Center, Atlantic City International Airport, NJ 08405 USA

**Keywords:** Engineering, Statistics

## Abstract

The verification of trace explosives detection systems is often constrained to small sample sets, so it is important to support the significance of the results with statistical analysis. As binary measurements, the trials are assessed using binomial statistics. A method is described based on the probability confidence interval and expressed in terms of the upper confidence interval bound that reports the probability of successful detection and its level of statistical confidence. These parameters provide useful measures of the system’s performance. The propriety of combining statistics for similar tests—for example in trace detection trials of an explosive on multiple surfaces—is examined by statistical tests. The use of normal statistics is commonly applied to binary testing, but the confidence intervals are known to behave poorly in many circumstances, including small sample numbers. The improvement of the normal approximation with increasing sample number is shown not to be substantial for the typical numbers used in this type of explosives detection system testing, and binary statistics are preferred. The methods and techniques described here for testing trace detection can be applied as well to performance testing of explosives detection systems in general.

## Introduction

Explosives trace detection employs chemical and spectroscopic techniques to screen passengers, baggage, and cargo for extremely small amounts of explosives^1,2^. When assessing performance of explosives trace detection systems or similar pass/fail devices, it is critical to utilize experimental data sets of sufficient size in order to effectively determine detection probabilities. Distinctions must be made between the observed alarm rate and the probability of detection to ideally describe the detector’s performance. The former, although correctly reporting the performance within the confines of the experiment, does not account for the probability that repeat trials with the same system under identical conditions may produce superior or inferior results. Presented here is the background on how the probability of detection is determined and also what impacts this parameter has on test and evaluation activities.

When deployed in screening environments, trace systems and other explosives detection systems^3,4^ (EDS) are binary in their operation. Trials conducted as binary measurements (i.e., “detection/alarm” or “no detection/no alarm”) can be assessed using binomial statistics after the method originally put forth by Clopper and Pearson^5^, who defined an interval for the probability of detection to a given degree of confidence. In this sense, the confidence interval is a measure of the repeatability of performance, and the probability is treated as a parameter that models the number of successful detections in multiple trials^6,7^. This differs from approaches to binary testing that evaluate the rate of occurrence of a characteristic in a population, such as in epidemiological studies^8,9^ or product conformance^10,11^ and product quality^12^; these treatments use probability of occurrence as a random variable^13,14^, often assuming the system pass rate is known^12,15^. While there is extensive literature on binary testing, the validation of trace explosives detection systems in particular is affected by the small number of samples used in trials, and how risk is assigned to the estimation of detection error (cf. Ref.^7^). In this paper, the binomial probability distribution function as applied to explosives detection systems is explained, and the probability of detection is presented in the context of the observed alarm rate.

To the point of testing to determine whether a system can meet an explosives detection requirement, we advocate using a one-tailed probability interval rather than the centralized interval^5,16^. The latter defines the confidence interval bounding the probability of detection to exclude as unlikely both high and low probability values. By applying an upper confidence limit only, as we describe here, we are specifically addressing the risk of overstating the probability. The chance that the probability will be overestimated is 1 minus the given confidence level.

Although the methodology in this work is based on discrete statistics, sequential testing methods have encompassed continuous models^2,17^. Sequential testing to quantify the limit of detection (LOD) of trace detection systems is standardized in ASTM E2677-20^18^. The testing standard explicitly cautions that proprietary signal processing can affect the Gaussian analysis of the LOD, and notes the security and classification issues that attend the LOD of explosives. The ASTM procedure requires the preparation of exploratory measurement samples to bracket the LOD for systematic statistical testing, with an optimum testing sample set of 4 $\times$ 12 samples, so potentially it has a greater experimental burden than a binary test. The binary testing of the instrument based on the methodology of this paper is viable for evaluating the suitability of a system for explosives screening, as appropriate or convenient. While comprehensive performance testing provides additional evaluation data^10^, screening at a checkpoint must distill all the ancillary information in the underlying signal—for example, through the application of filters and alarm thresholds^19^—into a binary pass or fail decision.

The treatment of the data collected in a binary test needs to ensure that statistically significant conclusions are drawn. When using relatively low numbers of experimental trials, high alarm rates do not necessarily equate to high probabilities of detection^16,20^, and the observed alarm rate and the probability of detection can be substantially different. As such, the validity of these results can be called into question. Increasing the sample size makes the discrepancy less. A properly-sized sample set can be facilitated by the multivariate approach routinely used with trace detection system testing, where multiple explosives are assessed on multiple surfaces using multiple masses. Combining sets of data can increase the sample size, but care must be taken to balance time required for testing with having enough data on each unique combination of test variables to maintain the minimum required statistical significance. The test data example presented in this paper describes an outcome of a system test using ten trials each on two different explosives and three substrate materials. The end goal of the analysis is to determine the probability of detection that conforms to the experimental alarm rate and can be statistically assured at a specified level of confidence.

Finally, we stress the importance of using binomial statistics for evaluating the test when the detection probability is high and when the sampling number is minimal by design. Specifically, it is not appropriate to approximate the binomial with a normal (Gaussian) distribution, which is often done to reduce the problem to the Wald test^13,21^. The relative error in this approximation as a function of the typical sample sizes for trace detection testing does not indicate for its use in place of binary statistics, as discussed in “Results”.

The methods and techniques described for testing trace explosives detection can be applied as well to performance testing of EDS and other detection technologies^3,22,23^.

## Methodology

### Relating the binomial distribution to explosive detection

**Figure 1:**
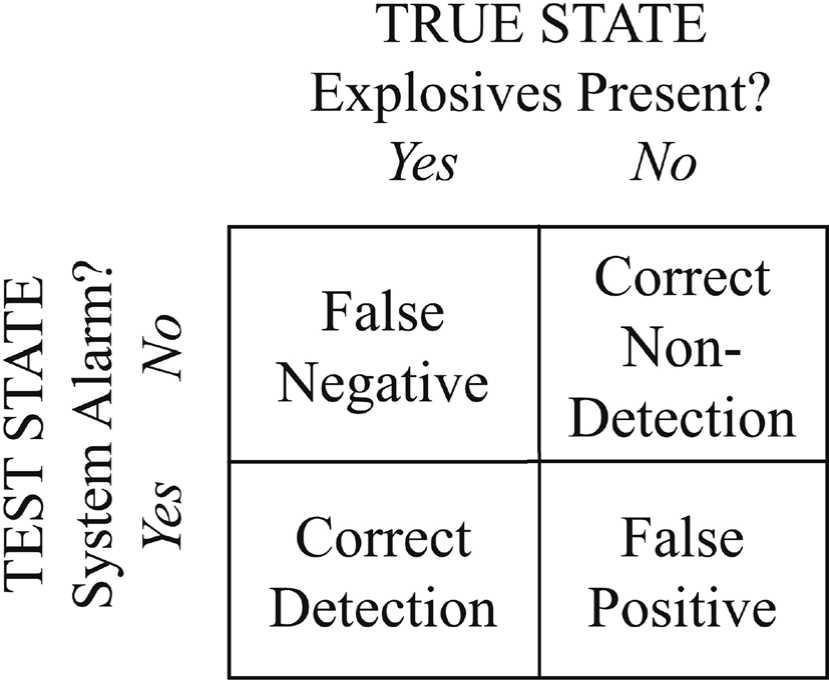
Response matrix of an explosives detector showing four possible outcomes.

For a system to be classified as binomial in its response, the following criteria must be met: the provided data is categorized as either a success or a failure, the probability of a success in a single trial is a constant factor throughout the experiment, and that all trials are independent, meaning the outcome of one trial does not affect subsequent outcomes. A binomial response of an explosive detector is illustrated in the classical representation^24–27^ in Figure 1.

Outcomes of binomial systems are governed by the binomial probability distribution function which is defined in the following relation where *p* is the probability of a successful detection in a single trial, *n* is the number of trials, and *x* is the number of successes

1 $$\begin{aligned} P(n,x,p)=\frac{n!}{x!\, (n-x)!}\, p^x \, (1-p)^{n-x} \end{aligned}$$

The probability function (*P*) provides an overall probability for a given number of successful detections when the probability is (*p*). For example, in flipping a coin it is understood that the probability *p* of heads (or tails) is 50%, but in small sample sets the number of heads or tails is known to be much different from 50%. Equation (1) describes the overall probability for a given outcome: for $n = 10$ flips, the most likely outcome for flipping a coin 10 times is indeed 5 heads (and 5 tails), but this is only expected 25% of the time.

The same principle exists when examining the detection capability of an explosives detector. The application of the binomial distribution is required as simple alarm rate data are not indicative of the true response of a system. For example, if 20 samples are presented to a detector and 18 return a positive alarm, it is correctly stated that the alarm rate is 90%. However, this need not accurately represent the true response features in an overall sense owing to the relatively small sample set. If this test were repeated, a higher or lower alarm rate might be observed by pure chance. To report instrument performance in the context of this variability, a useful approach used for binary testing is to specify the probability of detection (*Pd*) and an associated confidence level. These data carry more weight and meaning for the performance of an explosives detection system than the alarm rate observed in a test. *Pd* represents the most reliable estimate of *p* given the limited number of trials, and can vary depending on the risk accepted in estimating the probability. Thus, *Pd* changes depending upon the specified confidence level, but approaches *p* for large sample numbers. The application to detection systems will be illustrated by examining the impact of probability distributions on the detection confidence levels.

**Figure 2:**
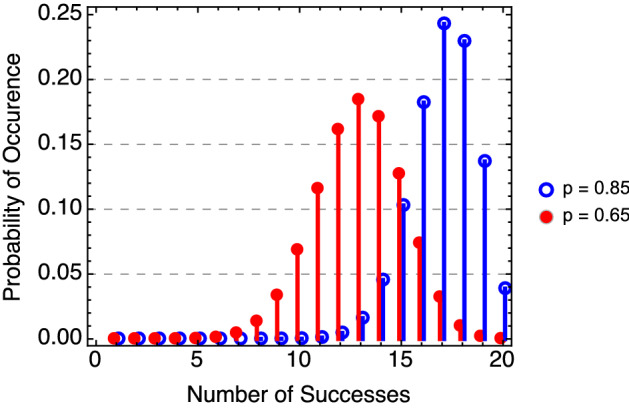
Binomial probability distribution functions for two values of *p* at $n=20$.

For this example, assume that $n=20$. Different values for probability *p* are illustrated with the binomial distributions in Fig. 2.

Dramatic differences in the distributions are seen between a system with a probability of success at 65% and another at 85%. For the latter, the probability of obtaining 20 successful trials in 20 attempts is only 4%. It is more likely that 17 or 18 successes will be observed. For $p=0.65$, it is clearly evident that the probability distribution has shifted to the left (13 successes is most likely). Here, there is only a 0.02% chance that 20 successful detections are observed. Determining the results in this fashion is straightforward when there is an understood or even theoretical value of *p*, such as for coin tossing. However, for explosives detection systems the probability that a single trial will result in a successful outcome is not known. The very reason for the test and evaluation process is to determine this value by examining responses across a series of threats and substrates with varied masses.

**Figure 3:**
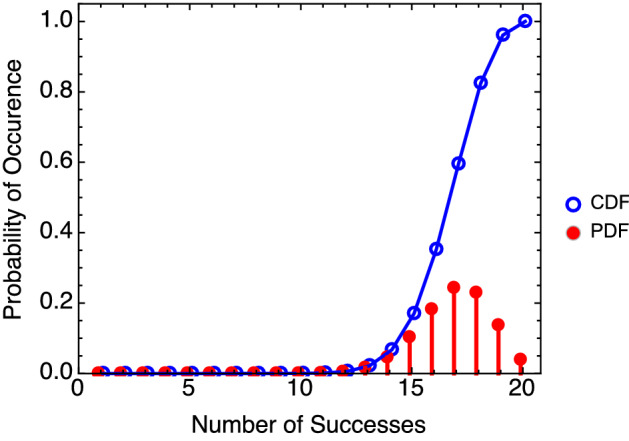
The binomial probability distribution function (PDF) and its cumulative distribution function (CDF) with $n=20$ and $p=0.85$.

The detection probability is found by an experiment that records *X* successes in a test of *n* samples. To determine *Pd*, the quantity of a confidence interval can be used. It is of no consequence if the explosives detector performs better than predicted, so our approach focuses on the upper confidence interval bound^6,21^. The confidence interval is defined through the cumulative binomial distribution (see Fig. 3), which is the sum of occurrence probabilities in detection number.

The parameter *p* is varied until the cumulative probability of detections at the numbers less than *X* encompasses a cumulative probability of $1-\alpha$. (Here, the parameter $\alpha$ also corresponds to the rate of false positives in Fig. 1). The probability value discovered in this way is regarded as the detection probability, $p=Pd$, at the confidence level $1-\alpha$. Explicitly, in terms of *n*, $\alpha$, and *X*, the solution for *Pd* is

2 $$\begin{aligned} \sum _{x=X}^n P(n,x,Pd) = \alpha . \end{aligned}$$

The cumulative distribution function returns the probability of a variable having a value less than or equal to the critical target value. This property can be exploited to determine the critical value of successes needed to establish a probability of detection to a predetermined level of confidence. Examples are given in Tables 1 and 2: for a number of values of *Pd*, the critical value *X* is listed for which the cumulative distribution is at least 90% of the total *n* in Table 1, and 95% of the total *n* in Table 2. The 90% and 95% define two confidence levels. Tables of confidence levels can be readily generated using computational tools. Values here are computed in Microsoft Excel as CRITBINOM$(n,p,CL)+1$. Given values *n* and *p*, CRITBINOM returns the smallest integer value for which the cumulative distribution is greater than or equal to $CL=1-\alpha$. The solution is one less than *X* in Eq. (2), so one integer is added. A calculator for *Pd* is provided in an electronic supplementary file.

**Table 1:** Binomial distribution critical values to establish detection probability at 90% confidence level.

Sample size	Probability *Pd*						
	65%	70%	75%	80%	85%	90%	95%
5	–						
10	9	10	10				
15	13	14	14	15	15		
20	17	18	18	19	20		
30	24	25	26	28	29	30	
50	38	40	42	45	47	49	50

**Table 2:** Binomial distribution critical values to establish detection probability at 95% confidence level.

Sample size	Probability *Pd*						
	65%	70%	75%	80%	85%	90%	95%
10	10	10					
15	14	14	15	15			
20	17	18	19	20	20		
30	25	26	27	28	29	30	
50	39	41	43	45	47	49	
60	46	49	51	54	56	59	60

The tables show the minimum number of successes for a given sample set size required to meet various probabilities of detection with the associated level of confidence. There are numerous implications regarding sample size and interpretation of results. For example, from Table 1 an alarm rate of 18 successes in 20 trials is considered a 75% probability of detection with 90% confidence. More accurately stated, the probability of detection is greater than or equal to 75% with 90% confidence; there is a 10% risk that the probability of detection is less than 75%. Finally, comparing Tables 1 and 2 it is evident that testing to a higher confidence level requires a larger sample size: for example, to demonstrate a Pd of 95% requires 60 test samples at the 95% confidence level versus 50 at the 90% confidence level.

### Relating the normal distribution to explosive detection

In the limit of large sample number, the binomial distribution can be approximated to the normal Gaussian distribution. This has been historically attractive owing to the more straightforward calculation of the probability function^21^, despite the computation of each being equally facile with modern computers. To illuminate the insufficiency of the normal approximation in the context of small sample number, the relative differences in the statistics will be considered.

The normal distribution is approximated to the binomial in the following manner:

3 $$\begin{aligned} f(x)=\frac{1}{\sigma \sqrt{2 \pi }} \, e^{-(x-\mu )^2/2 \sigma ^2 } , \end{aligned}$$

where the standard deviation $\sigma$ is given by

4 $$\begin{aligned} \sigma =\sqrt{np(1-p))} \end{aligned}$$

and $\mu$ is the mean of the distribution given by

5 $$\begin{aligned} \mu =n p . \end{aligned}$$

This relationship can be used to construct a Table  3 analogous to Table 1 but based on the normal distribution.

**Table 3:** Normal distribution critical values to establish detection probability at 90% confidence level.

Sample size	Probability *Pd*						
	65%	70%	75%	80%	85%	90%	95%
5	5	5	5				
10	9	9	10	10	10		
15	13	13	14	14	15	15	
20	16	17	18	19	20	20	
30	23	25	26	27	29	30	
50	37	40	42	44	46	48	50

It can be seen when comparing selected success rates between Tables 1 and 3 that the *Pd*’s generated by the latter are more lenient. For instance, 10 successes in 10 trials equates to a *Pd* greater than or equal to 75% at the 90% confidence level when determined with the binomial. The same criteria result in a *Pd* of 85% with the normal approximation. This error illustrates the inadequacy of the normal approximation in assigning confidence limits to detection probabilities.

## Results

The testing method outlined above can establish whether the detection probability of an explosives detection system meets a required performance standard at a specified confidence level. As discussed previously, *Pd* better mirrors the measured alarm rate when the experimental sample set is large. When the sample size is small, the ability of *Pd* to discern high detection rates is diminished, and the the demonstrated *Pd* can be much less than the actual detection probability *p*. In order to avoid lengthy tests to meet *Pd* requirements for sample size, trials with an overlapping variable may be combined to create a larger sample set thereby increasing the effective sample size.

The ability to combine data sets is dependent upon the responses of the system. In trace detection test and evaluation studies, explosive residues are dry transferred^28^ to standardized substrates to mimic field screening scenarios in the laboratory. Routinely, multiple explosives are tested on several substrates, creating a test matrix. The larger the test set, the greater the predictive power of performance in the field, but a balance is needed between testing breadth and necessary resources. Consider hypothetical outcomes of a trace system test shown in Table 4. In this example the numbers of threats are minimal in scope to permit for a more straightforward analysis. Note that for Explosive 1 on Substrate A, 10 of 10 trials returned successes to establish a probability of detection *Pd* for this experimental set at 75% with 90% confidence.

**Table 4:** Explosive trace detector (ETD) collected data.

Explosive	Substrates			Totals
	A	B	C	
1	10/10	10/10	9/10	29/30
2	9/10	9/10	4/10	22/30
Totals	19/20	19/20	13/20	51/60

Given the similar data with other surfaces, the statistics of Explosive 1 on all three surfaces can be combined for a success rate of 97% (29 of 30 successes), equivalent to a *Pd* of 85% with 90% confidence. Combining such statistics is permissible when similar detection trends are observed for a given explosive at a given mass on all three surfaces, i.e. the variable of test substrate does not affect alarm rates. This applies for Explosive 1, but not for Explosive 2 where Substrate C contributed to poor alarm rates. As such, these data should not be combined with the Explosive 2 data on the other two substrates.

To determine when it is appropriate to combine such data, hypothesis testing can be used to compare the individual binomial distributions. The null hypothesis in this case states that there is no statistical difference in any of the surface detection data. If rejected through statistical analysis, the alternate hypothesis that the test values vary with surface material must be accepted. The Explosive 2 data above for all three surfaces can be analyzed using Fisher’s Exact test, which is an ideal test for data having small numbers of replicates. Table 5 demonstrates the input prepared for a $2 \times 3$ Fisher Test on the Explosive 2 data. Calculation gives a low probability ($p=0.022$) that the data for the three surfaces are statistically equivalent. Further statistical analysis—such as Pearson’s chi-square test—can be applied to confirm where the differences originate.

**Table 5:** Explosive #2 data formatted for Fisher’s Exact Test determination.

	A	B	C	Total
Hits	9	9	4	22
Misses	1	1	6	8
Totals	10	10	10	30

Given the result of the equivalence test, not all values can be combined to form an effective larger sample set, and it is not statistically valid to utilize a success rate of 51/60 to determine a *Pd* for the entire trial. It is acceptable to assess the performance of the EDS on Explosive 1 across all substrates. For Explosive 2, only Substrates A and B may be combined; Substrate C must be reported separately. For this reason, sample sizes are encouraged such that individual data points have statistical weight in the event they cannot be combined with other data of the same threat.

A consequence to using small sample sets to assess performance is the gap between alarm rate and the assessed *Pd*. As an example, three out of three successes (100% alarm rate) does not assure that the system performs even at the detection rate *Pd* of 50%. As apparent from Tables 1 and 2, larger sample size improves the likelihood that the observed alarm rate will mirror the determined *Pd*. While the minimum sample size is what is necessary to determine the *Pd* within the requirements, the minimum number anticipates 100% alarm rate, and the gap between the alarm rate and the detection rate means that the specified *Pd* may not represent the capability of the system. As such, it is still beneficial to keep the sample size as large as practical when conducting test and evaluation studies.

The application of the significance interval can justify abandoning a test when the number of false negatives precludes establishing the *Pd* at the desired significance. The number of ‘misses’ that can be tolerated to maintain the desired detection probability is based on the cumulative distribution function. In Table 6, the number of allowed misses is $n - X$, where *X* is the number of successes required at the specified *Pd* and confidence level (i.e., the computed quantity in Table 1).

**Table 6:** Maximum number of allowed misses ensuring *Pd* at the 90% confidence level.

Sample size	Probability *Pd*						
	65%	70%	75%	80%	85%	90%	95%
10	1	0	0				
15	2	1	1	0	0		
20	3	2	2	1	0		
30	6	5	4	2	1	0	
50	12	10	8	5	3	1	0

While the data above are geared toward the 90% level of confidence, any level can be substituted and the results utilized per the testing laboratory’s discretion. Importantly, the desired confidence level and *Pd* must be agreed upon before conducting testing. As one would expect, the greater the value of *Pd* and confidence level, the greater the number of replicates required to meet these criteria.

In order to guard against the possibility of false positives (see Fig. 1), control samples with no explosives are tested to evaluate the probability of correctly determining the absence of explosive^29^. This data set can be quantified in the same way as with the explosive sample set, with the reasonable assumption that the measurements are conditionally independent^12,30^. While the detection significance is quantified by the false negative rate $\alpha$, the corresponding significance level of false positives is set by the error rate $\beta$. The limits on these errors vary widely with application^31,32^.

For explosives detection systems used in security environments, the requirements for system errors are ultimately established by governmental or regulatory authorities. The significance level associated with false negatives must be small to minimize the risk of admitting an explosive threat. While the false positive rate in screening is less critical because all positive alarms are generally subject to additional evaluation^8^, false positives at checkpoints still come with the costs of the secondary screening and the undesired consequence of causing delays^33^. (An interesting aspect to the problem is how likely it is that a system alarm is actually identifying an explosive^9,12,34,35^. This probability depends on factors in addition to the significance level $\alpha$ of the detection, and includes the prior probability that an explosive occurs (i.e., the ‘odds’ that an explosive is present), and the statistical power $1-\beta$).

**Figure 4:**
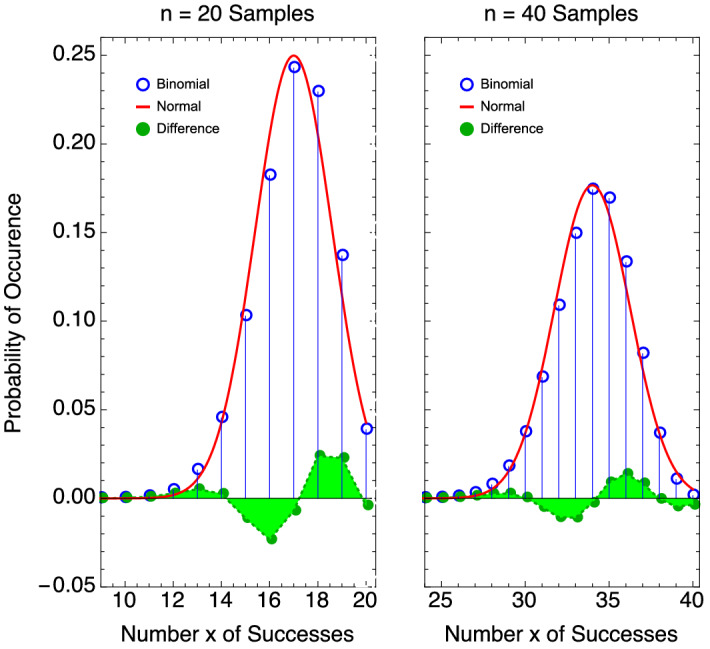
Binomial and normal probability distributions with $p=0.85$ for different sample size: left, $n=20$ and right, $n=40$. The error in the binomial approximation is shown for each case.

**Figure 5:**
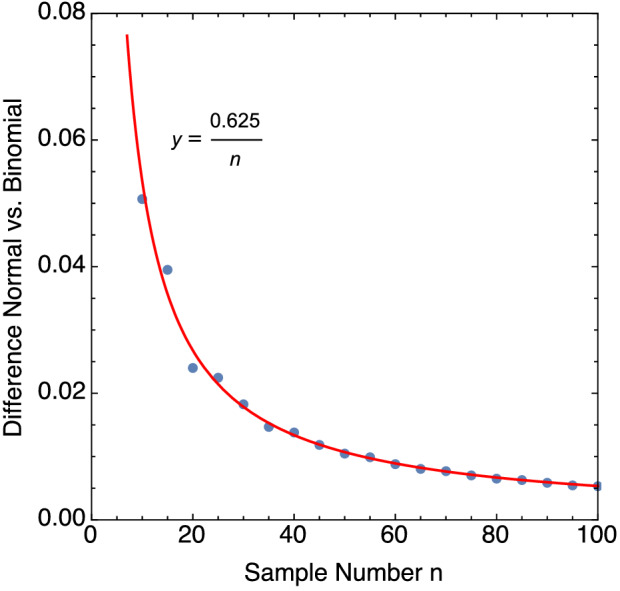
Difference in binomial and normal distributions as a function of sample size *n* for $p=0.85$. The mismatch in probability diminishes as the sample size gets larger, but the fitted curve shows the error decreases weakly with n.

On the issue of the propriety of using the normal distribution to define the confidence level, we examine the inherent error in the approximation to binomial statistics. In Fig. 4 the normal and binomial distributions are plotted for identical detection probability and different sample numbers. The figures show that the error in the normal approximation is somewhat less in the case of the larger sample size. The raw difference in the probabilities between the normal and binomial distributions are plotted for a wide range of sample sizes and $p=0.85$ in Fig. 5.

The raw differences display the power decay function with sample size suggested by the fitted curve 0.625/*n* in Fig. 5. It is clear that the normal approximation does not improve rapidly with sample number; it is also known to behave poorly in many circumstances^13^. Our view is that the error in the approximation illustrated in Fig. 5 does not argue for adopting the normal distribution approximation with the minimal sample numbers illustrated here for explosives testing. Alternative probability intervals that are more accurate include the Wilson interval^36^, Agresti-Coull^21^ interval, and the Jeffreys interval (based on Bayesian prior beta distribution^7,13^), but for the purpose of small number sampling the binomial interval seems the best choice based on its simplicity and suitability.

## Conclusion

Utilizing a probability of detection with an associated level of confidence is a more statistically robust metric to describe instrument performance than the alarm rate. The latter are meaningful, but only in the confines of the experiment conducted. Repeat trials, or trials outside the boundary of the experimental conditions, will undoubtedly yield different results whereas *Pd* is meant to encompass more information about the system performance than the experiment outcome itself.

The desired levels of *Pd* and confidence level must be set before testing is begun. In order to assure the system under test meets these criteria, the sample size cannot be too small. The minimum sample size that can support the criteria requires all successful detections, but larger sample sizes allow detection rates—in particular, detection failures—that are more representative of the system. For a given explosive on a single substrate, to achieve a *Pd* of 80% at the 90% confidence level requires 15 trials, with all 15 successful detections. Two or three substrates are routinely tested with trace analysis, offering the possibility to effect sample sizes of 30 or 45, assuming similar success rates are observed across the substrates. With 30 samples, a *Pd* of 80% can be accepted with 2 misses, while for $n=45$, 5 misses can be allowed.

Owing to the relatively limited sample sizes anticipated for trace analysis, the normal approximation for probability interval calculation should be avoided. Implementing the binomial distribution is not only more accurate than the normal distribution for small samples sizes, but also embodies the underlying statistics describing the response probability of trace detectors.

In summary, this paper presents a methodology for specifying the detection rate of an explosives detection system, and promotes a testing standard to validate that systems meet EDS requirements. The definition of probability and confidence level derives from a one-tailed probability interval of the binomial distribution. Common use of the probability interval is two-tailed, but in the application to explosives detection, the emphasis to avoid overstating the detection probability favors using the one-tailed cumulative probability. Because trace explosives testing is often handicapped with small sample numbers, a statistical test to justify combining similar sample sets is described as a way to increase the sample number and enhance the statistical significance of the test.

## Supplementary Information

Supplementary Information.
